# Insomnia Really Hurts: Effect of a Bad Night's Sleep on Pain Increases With Insomnia Severity

**DOI:** 10.3389/fpsyt.2018.00377

**Published:** 2018-08-28

**Authors:** Yishul Wei, Tessa F. Blanken, Eus J. W. Van Someren

**Affiliations:** ^1^Department of Sleep and Cognition, Netherlands Institute for Neuroscience, An Institute of the Royal Netherlands Academy of Arts and Sciences, Amsterdam, Netherlands; ^2^Department of Integrative Neurophysiology, Center for Neurogenomics and Cognitive Research, Amsterdam Neuroscience, Vrije Universiteit Amsterdam, Amsterdam, Netherlands; ^3^Department of Psychiatry, Amsterdam UMC, Vrije Universiteit Amsterdam, Amsterdam, Netherlands

**Keywords:** sleep quality, pain, insomnia disorder, somatic complaints, symptom dynamics, reactivity, sensitization

## Abstract

Insomnia and chronic pain are highly prevalent conditions and are often comorbid. Somatic complaints other than pain are also often observed in insomnia. Poor sleep and pain are known to mutually reinforce each other. However, it is unknown whether the habitual severity of insomnia modulates the acute effect of a particularly bad night's sleep on the next day's pain severity, and whether it modulates the acute effect of pain on the following night's sleep quality. Using data from 3,508 volunteers (2,684 female, mean age 50.09 y), we addressed these questions in addition to the associations between the habitual severity of insomnia, somatic complaints, and pain. Results indicated that people suffering from more severe habitual insomnia showed stronger mutual acute within-day reactivity of pain and poor sleep quality. The same increased reactivity was found in people with more severe habitual pain. Interestingly, the acute within-day mutual reactivity of pain and sleep quality showed consistent asymmetry. Pain worsened more after a particularly bad night's sleep than it improved after a particularly good night's sleep. Likewise, sleep worsened more after a day with more-than-usual pain than it improved after a day with less-than-usual pain. Future interventions may profit from addressing this asymmetric mutual reactivity especially in people with severe comorbid insomnia and chronic pain.

## Introduction

Insomnia is the most prevalent sleep disorder and the second most prevalent mental disorder in Western countries (1). The prevalence of insomnia estimated by epidemiological studies ranges from 6% to one third, depending on the definition of insomnia and the source population (2). Furthermore, insomnia represents an important risk factor for the development of various medical conditions, including cardiovascular diseases (3, 4), diabetes (5), and other mental disorders (6).

Insomnia is often observed to accompany somatic complaints, including pain. Between 50 and 88% of people with chronic pain who seek medical help also complain about insomnia (7, 8) and, vice versa, at least 40% of people with insomnia have chronic pain (9, 10). Disrupted sleep further predicts the frequency (11), extent (12), and intensity (13) of pain in the general population. Other somatic complaints besides pain have also been linked to insomnia, although such associations have been studied less extensively (14–16).

It is generally accepted that the relationship between pain and insomnia is reciprocal (17, 18). Recent large-scale longitudinal population-based studies have convincingly shown that insomnia predicts new incidence of chronic pain (19, 20) and vice versa (21, 22). Among patients with chronic pain, insomnia symptoms not only are associated with greater pain intensity, pain-related disability, and depression (23, 24), but also have impacts on prognosis (25–28). A limitation of these studies on the consequences of insomnia on pain, already acknowledged by some authors, is that insomnia is almost always assessed with self-reports on sleep problems, while daytime impairments which are part of the defining characteristics of insomnia (29, 30) have typically not been taken into account.

Although the above-mentioned observational studies provide compelling evidence for the impacts of disrupted sleep on pain, interventional studies often find that treatments targeting insomnia only contribute to small reduction of pain in patients with comorbid insomnia and chronic pain (31, 32). The reasons for this discrepancy between observational and interventional studies are at present insufficiently understood. It could be that improved sleep quality alleviates pain to a less extent than insomnia aggravates pain in general. Alternatively, the observed associations between pain and sleep quality may be confounded or modulated by other factors. Systematically studying individual differences in the reactivity profile with respect to pain and sleep quality may provide clues about the factors involved.

The current study aims at depicting a clearer picture of the relationship between sleep quality and pain along these lines. Using data from a psychometric database contributed by volunteers in the community, we first answer the question, “Do painful conditions improve after a particularly good night's sleep to the same degree as they worsen after a particularly bad night's sleep?” and the corresponding question regarding the reverse effect of pain on sleep. We next explore the possibility that a person's reactivity to poor sleep in terms of pain severity might depend on the baseline insomnia severity. In other words: Does a bad night's sleep increase the next day's pain to the same degree in people with habitual poor sleep as it does in people with habitual good sleep? Likewise, one might ask whether painful experience affects subsequent sleep to the same degree in people with habitual poor sleep as it does in people with habitual good sleep. The same two questions can be asked for people with less vs. more habitual pain. We complement the investigation with assessments of associations of habitual insomnia severity with the overall severity of habitual pain and somatic complaints. To our knowledge, this is the first study to investigate how mutual vulnerabilities between acute pain and disturbed sleep may be modulated by habitual pain and disturbed sleep.

## Materials and methods

### Participants

Questionnaire data were obtained through the Netherlands Sleep Registry (NSR), an online platform for psychometric data collection across the general population. Volunteers for the NSR include good and poor sleepers recruited through advertisement on the internet, television, radio, magazines, and newspapers as well as through flyers distributed in health care institutions and conventions. Each participant registered for an account on the NSR website (www.sleepregistry.nl) and could then complete a wide array of survey modules online concerning demographics, personality, psychosocial well-being, life events, and medical history. Each of the online survey modules contains one or more questionnaires, as detailed previously (33). Because the sheer number of surveys precluded the possibility of completing all modules in one sitting, participants were allowed to fill them out bit by bit at self-chosen times and places. As a result, a different number of participants had completed each individual survey module at the time of analysis. The current analyses utilized data entered between January 2011 and October 2017 by a total number of 3508 participants. No exclusion criteria other than missing of questionnaire data necessary for the subsequent analyses was imposed. The ethics committee of the Academic Medical Center, University of Amsterdam, Amsterdam, The Netherlands, and the Central Committee on Research Involving Human Subjects, The Hague, The Netherlands, approved of unsigned informed consent because volunteers participated anonymously without revealing their full names and addresses and were not exposed to any intervention or behavioral constraint.

### Measures

#### Insomnia severity index (ISI)

The Insomnia Severity Index (34) is a seven-item Likert scale measuring the severity of nighttime and daytime symptoms of insomnia. Each item is a 5-point rating (0–4) concerning a distinct aspect of insomnia over a 2-week period, denoted hereafter as “habitual.” The total sum score, ranging from 0 to 28, provides an overall index of insomnia severity. The ISI has been shown to have good psychometric properties, including high diagnostic accuracy (34–36).

#### Four-dimensional symptom questionnaire (4-DSQ)

The Four-Dimensional Symptom Questionnaire (37) comprises 4 subscales: distress, somatization, anxiety, and depression. Each subscale measures the severity of a suite of psychological symptoms over a 1-week period, denoted hereafter as “habitual.” The questionnaire has been designed so that the overlap between subscales is minimal. That is, symptoms specific to depression, anxiety, and somatic complaints are captured by the corresponding subscales, while the distress subscale assesses non-specific symptoms reflecting the general level of mental distress. The ranges of the subscales are, respectively, 0–32 for distress, 0–32 for somatization, 0–24 for anxiety, and 0–12 for depression. The 4-DSQ has been shown to have good psychometric properties, including high diagnostic accuracy (37–40).

#### Chronic pain grade (CPG)

The Chronic Pain Grade (41) assesses pain severity across a period up to 6 months, denoted hereafter as “habitual”. It provides 3 quantitative scores, i.e. pain intensity, pain-related disability, and days with pain-related disability. For clinical purposes the scores can be combined to determine a final categorical grade (5 levels from pain-free to high disability–severely limiting). Given the aim of the current study, only the pain intensity score (range: 0–100) was used.

The pain assessment module of the NSR also contains 4 items about the perceived acute relationship between sleep and pain, similar to those used in a previous study (42). The perceived acute effect of sleep on pain is addressed by 2 items, “If I sleep worse/better than usual on one night, the next day the chance of feeling pain is …” and the answer options are “much smaller,” “smaller,” “somewhat smaller,” “as usual,” “somewhat bigger,” “bigger,” and “much bigger” (coded as −3, −2, −1, 0, 1, 2, and 3, respectively). The perceived acute effect of pain on sleep is addressed by 2 items, “If I have a day with more/less pain than usual, the following night I usually sleep …” and the answer options are “much worse,” “worse,” “somewhat worse,” “as usual,” “somewhat better,” “better,” and “much better” (coded as −3, −2, −1, 0, 1, 2, and 3, respectively).

### Statistical analyses

We first calculated Spearman correlation coefficients to examine the associations of habitual insomnia severity with the habitual severity of somatic complaints and habitual pain intensity. Next, linear regression was employed to control for possible effects of age, sex, and the other 4-DSQ subscales.

Data on perceived acute within-day sleep–pain relationship were analyzed in 3 steps. First, Wilcoxon signed-rank tests were performed to evaluate whether the central tendency of each rating significantly deviated from the 0 rating that indicates “as usual,” i.e., no effect. Second, two Wilcoxon signed-rank tests ensued to compare (1) the absolute acute effect of worse-than-usual sleep with that of better-than-usual sleep on subsequent pain, and (2) the absolute acute effect of more-than-usual pain with that of less-than-usual pain on subsequent sleep. Finally, Spearman correlation coefficients were used to investigate whether habitual insomnia severity and habitual pain intensity modulated the strength of the within-day mutual reactivity of pain and sleep quality. In addition to statistical significance, the robustness of the correlations was further verified by their 95% bootstrap confidence intervals (computed over 10,000 resampling iterations).

## Results

The distributions of age, sex, ISI, 4-DSQ subscales, CPG pain intensity score, and ratings on perceived acute sleep–pain relationship are presented in Table 1. Of all participants, 2497 completed the 4-DSQ, 2873 completed the CPG together with ratings on perceived acute sleep–pain relationship, and 1862 completed all of the questionnaires. People who completed only the CPG did not differ from those who also completed the 4-DSQ in terms of habitual pain or insomnia severity (Table 2). People who completed only the 4-DSQ had less severe habitual insomnia and somatic complaints as compared to those who also completed the CPG. The difference in habitual insomnia severity was secondary to the difference in sex distribution as the ISI within each sex did not differ between the subsamples (results not shown). In contrasts, the differences in somatic complaints between subsamples remained significant even when comparisons were performed separately for each sex (results not shown).

**Table 1:** Characteristics of participants (mean ± standard deviation).

Age (y)	50.09 ± 15.24
Sex (female/male)	2684/824
ISI	10.12 ± 7.17
**4-DSQ (*N* = 2,497)**	
Distress	10.08 ± 8.12
Somatization	7.11 ± 5.62
Anxiety	2.25 ± 3.65
Depression	1.50 ± 2.84
**CPG pain intensity (*N* = 2,873)**	40.42 ± 20.08
**Perceived sleep–pain relationship (*N* = 2,873)**	
Pain after worse sleep^a^	0.52 ± 0.82
Pain after better sleep^a^	−0.38 ± 0.76
Sleep after more pain^b^	−0.50 ± 0.97
Sleep after less pain^b^	0.18 ± 0.63

*4-DSQ, Four-Dimensional Symptom Questionnaire; CPG, Chronic Pain Grade; ISI, Insomnia Severity Index*.

a *Responses to the items “If I sleep worse/better than usual on one night, the next day the chance of feeling pain is …” with ratings ranging from −3 (much smaller) to 3 (much bigger)*.

b *Responses to the items “If I have a day with more/less pain than usual, the following night I usually sleep …” with ratings ranging from −3 (much worse) to 3 (much better)*.

**Table 2:** Characteristics of participants (mean ± standard deviation) within subsamples according to questionnaires completed.

	**CPG + 4-DSQ (*N* = 1,862)**	**CPG only (*N* = 1,011)**	**4-DSQ only (*N* = 635)**
Age (y)	50.83 ± 14.46	48.81 ± 16.67^*^	49.96 ± 14.97
Sex (female/male)	1459/403	782/229	443/192^***^
ISI	10.17 ± 7.10	10.36 ± 7.16	9.61 ± 7.41^*^
**4-DSQ**
Distress	10.34 ± 8.06	–	9.33 ± 8.23^***^
Somatization	7.59 ± 5.59	–	5.72 ± 5.49^***^
Anxiety	2.35 ± 3.72	–	1.95 ± 3.44^**^
Depression	1.53 ± 2.87	–	1.43 ± 2.73
**CPG pain intensity**	40.81 ± 19.86	39.70 ± 20.47	–
**Perceived sleep–pain relationship**			
Pain after worse sleep^a^	0.52 ± 0.82	0.51 ± 0.82	–
Pain after better sleep^a^	−0.38 ± 0.75	−0.40 ± 0.76	–
Sleep after more pain^b^	−0.50 ± 0.97	−0.49 ± 0.97	–
Sleep after less pain^b^	0.19 ± 0.64	0.18 ± 0.62	–

*4-DSQ, Four-Dimensional Symptom Questionnaire; CPG, Chronic Pain Grade; ISI, Insomnia Severity Index*.

*, **, *** Significance of difference from the “CPG + 4-DSQ” subsample (*p < 0.05, **p < 0.01, ^***^p < 0.001) as determined by Fisher exact test for sex and by Wilcoxon rank-sum tests for the other variables.

a *Responses to the items “If I sleep worse/better than usual on one night, the next day the chance of feeling pain is …” with ratings ranging from −3 (much smaller) to 3 (much bigger)*.

b *Responses to the items “If I have a day with more/less pain than usual, the following night I usually sleep …” with ratings ranging from −3 (much worse) to 3 (much better)*.

Habitual insomnia severity (ISI) correlated with the somatization subscale of the 4-DSQ (Spearman correlation coefficient = 0.47, *p* < 0.001), and with the pain intensity score from the CPG (Spearman correlation coefficient = 0.33, *p* < 0.001). Regression coefficients are presented in Tables 3, 4, for models with the somatization subscale of the 4-DSQ as outcome and with the pain intensity score from the CPG as outcome, respectively. In Table 3 one sees that habitual insomnia severity was robustly positively associated with the habitual severity of somatic complaints, even after controlling for the effects of the other 4-DSQ subscales (depression, anxiety, and general distress). Similarly, in Table 4 one sees that habitual insomnia severity was robustly positively associated with habitual pain intensity, even after controlling for the effects of all 4-DSQ subscales (depression, anxiety, somatization, and general distress).

**Table 3:** Regression model with habitual severity of somatization / somatic complaints as outcome variable (*N* = 2,497).

	**Regression coefficient**	**Standard error**	***t***	***p***
(Intercept)	2.074	0.390	5.31	<0.001
Age (y)	−0.005	0.006	−0.79	0.43
Female	0.656	0.202	3.25	0.001
ISI	0.115	0.015	7.85	<0.001
4-DSQ Distress	0.302	0.020	15.46	<0.001
4-DSQ Anxiety	0.387	0.032	12.02	<0.001
4-DSQ Depression	−0.191	0.044	−4.34	<0.001

*4-DSQ, Four-Dimensional Symptom Questionnaire; ISI, Insomnia Severity Index*.

**Table 4:** Regression model with habitual pain intensity as outcome variable (*N* = 1,862).

	**Regression coefficient**	**Standard error**	***t***	***p***
(Intercept)	15.56	1.97	7.90	<0.001
Age (y)	0.18	0.03	6.01	<0.001
Female	3.25	1.02	3.20	0.001
ISI	0.50	0.07	6.91	<0.001
4-DSQ Distress	−0.12	0.10	−1.26	0.21
4-DSQ Somatization	1.29	0.10	13.41	<0.001
4-DSQ Anxiety	−0.21	0.16	−1.33	0.18
4-DSQ Depression	0.44	0.21	2.08	0.04

*4-DSQ, Four-Dimensional Symptom Questionnaire; ISI, Insomnia Severity Index*.

Table 1 also shows that, as expected, people reported to have more pain than usual after they experienced a particularly bad night's sleep and less pain than usual after they experienced a particularly good night's sleep. Wilcoxon signed-rank tests confirmed that these ratings significantly differed from 0 (both *p* < 0.001). Interestingly, the acute impact of changes in sleep quality on pain was not symmetric. A particularly bad night's sleep increased the next day's pain significantly more than a particularly good night's sleep ameliorated the next day's pain (mean difference ± standard deviation = 0.13 ± 0.63, *p* < 0.001).

Similarly, people reported to have significantly worse sleep than usual following a day during which they experienced more pain than usual (*p* < 0.001). They also reported to have significantly better sleep than usual following a day during which they experienced less pain than usual (*p* < 0.001). The acute impact of changes in pain on sleep quality was again not symmetric. A day with more pain than usual worsened the night's sleep quality more than a day with less pain than usual improved the night's sleep quality (mean difference ± standard deviation = 0.31 ± 1.01, *p* < 0.001).

**Figure 1:**
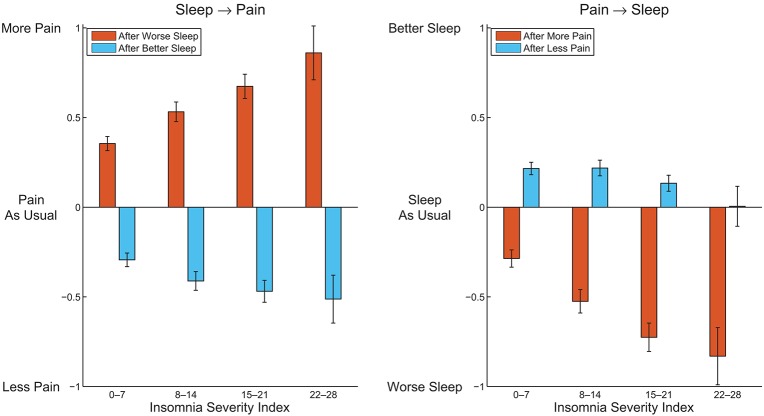
Average perceived pain after a better night and worse night of sleep than usual **(Left)**, and average perceived sleep quality after a day with more pain and less pain than usual **(Right)**, within subgroups of participants defined by clinical cutoffs of the Insomnia Severity Index. Error bars indicate 95% confidence intervals.

Remarkably, all of the 4 ratings on perceived acute sleep–pain relationship significantly correlated with the pain intensity score from the CPG, and 3 out from 4 significantly correlated with the ISI (Table 5). Figure 1 visualizes how the mutual acute within-day reactivity of pain and sleep quality was modulated by habitual insomnia severity. Participants are grouped according to clinical cutoffs of the ISI (34). The left panel of Figure 1 shows that the acute effect of sleep quality on the next day's pain increased in both directions with increasing habitual insomnia severity. The blue bars in the right panel of Figure 1 illustrate that the improvement in sleep quality after a day with less pain than usual was independent of habitual insomnia severity within the subclinical range (the two groups with ISI < 15), while in the clinical insomnia groups (ISI ≥ 15), the benefit for sleep due to a day with less pain than usual disappeared with increasing habitual insomnia severity. In strong contrast, the red bars in the right panel of Figure 1 illustrate that acute worsening of sleep after a day with more pain than usual continued to increase with more severe habitual insomnia.

**Table 5:** Spearman correlation coefficients between perceived acute sleep–pain relationship and habitual insomnia/pain severity (*N* = 2,873; brackets indicate 95% bootstrap confidence intervals).

	**Sleep**→**Pain**		**Pain**→**Sleep**	
	**Pain after worse sleep^a^**	**Pain after better sleep^a^**	**Sleep after more pain^b^**	**Sleep after less pain^b^**
ISI	0.19^***^ [0.16, 0.23]	−0.13^***^ [−0.17, −0.10]	−0.20^***^ [−0.23, −0.16]	−0.02 [−0.05, 0.02]
CPG pain intensity	0.19^***^ [0.15, 0.22]	−0.06^**^ [−0.10, −0.02]	−0.26^***^ [−0.30, −0.22]	0.10^***^ [0.06, 0.14]

*CPG, Chronic Pain Grade; ISI, Insomnia Severity Index*.

** *p < 0.01*,

*** *p < 0.001*.

a *Responses to the items “If I sleep worse/better than usual on one night, the next day the chance of feeling pain is …” with ratings ranging from −3 (much smaller) to 3 (much bigger)*.

b *Responses to the items “If I have a day with more/less pain than usual, the following night I usually sleep …” with ratings ranging from −3 (much worse) to 3 (much better)*.

## Discussion

The current study delineates the perceived acute within-day sleep–pain relationship in a community-based sample on top of the associations between habitual insomnia and habitual somatic complaints (including pain). We show, in addition to robust associations of habitual insomnia severity with the habitual severity of somatic complaints and pain, that people in general perceive reciprocal, but asymmetric, acute effects of incidental changes in sleep quality and pain severity on each other—with the effects of worse sleep and more pain than usual larger than those of better sleep and less pain than usual, respectively. Importantly, the strength of the perceived acute reciprocal effects is generally stronger in people with more severe habitual insomnia and pain, with the notable exception being that the benefit for sleep due to a day with less pain than usual is gradually lost with increasing habitual insomnia severity.

The associations of the perceived acute sleep–pain relationship with habitual insomnia severity and habitual pain intensity reported here are novel and may have clinical implications. It is known that people with insomnia exhibit larger night-to-night sleep variability than people without (43). This larger variability may be in part driven by larger fluctuations in the severity of physical symptoms, in line with a recent study showing that self-reported nocturnal wake time is associated with the fluctuation in pain over a week (44). Our results further refine this association by highlighting an asymmetric effect of the fluctuation in pain on sleep quality, especially for people with severe habitual insomnia or pain. In fact, in people with severe habitual insomnia, transient reduction of pain has virtually no effect on subsequent sleep quality (Figure 1). When it comes to treatment programs for patients with chronic pain, especially for those with severe comorbid insomnia, it therefore cannot be assumed that sleep problems would automatically be resolved upon alleviation of pain—a point already emphasized by other authors (45, 46). The acute effect of the fluctuation in sleep quality on pain is also asymmetric, and increases with habitual insomnia or pain severity in both directions. The increase is larger for worse-than-usual sleep than for better-than-usual sleep, resulting in even more exaggerated asymmetry in people with severe habitual insomnia or chronic pain (cf. Figure 1). This could possibly explain why previous interventional studies have reported only weak and inconsistent effects of treatments targeting insomnia on pain in patients with clinically comorbid insomnia and chronic pain (31, 32). Possible reactivity differences between people may therefore need to be carefully considered in future intervention development to achieve more efficient management of comorbid insomnia and chronic pain.

To our knowledge, the perceived acute sleep–pain relationship has only been investigated within a specific clinical sample in a smaller-scale study (42). Our results about the asymmetric perceived acute sleep–pain relationship agree with that study, thereby demonstrating generalizability. A handful of previous community-based studies investigated the relationship between habitual insomnia severity and habitual somatic complaints (14–16) and more specifically between habitual insomnia severity and habitual pain intensity (13, 47). However, the definitions of insomnia adopted by most of these studies referred only to night-time symptoms (difficulty initiating sleep, difficulty maintaining sleep, early morning awakening, nonrestorative sleep and/or poor sleep quality) whereas we here evaluated insomnia severity using the ISI which also assesses daytime impairments. Furthermore, different sets of psychological symptoms (or none at all) were controlled for in different studies, making comparisons somewhat difficult. The most similar study to the current one, but of a smaller scale, was conducted by Zhang et al. (15). That study reported robust associations of the ISI with the severity of pain and non-pain somatic complaints over the prior week, after controlling for the Hospital Anxiety and Depression Scale scores (48) which overlap in content with the distress, anxiety and depression subscales of the 4-DSQ. In conclusion, our result with respect to somatic complaints within 1 week can be regarded as corroborating the finding of Zhang et al. (15), whereas the result on pain intensity generalizes their finding to a longer reference period (up to 6 months).

The mechanisms underlying the relationship between insomnia and pain (or somatic complaints in general) are still not well understood (18, 49). It is known that short or disrupted sleep can acutely induce low pain threshold (50, 51). Chronic insomnia, in particular, is associated with hypersensitivity to interoceptive input which may involve heightened brain excitability, attentional bias, or deficient salience filtering (52, 53). The caudate nucleus, a subcortical structure known to be involved in pain suppression (54), has also been shown to be affected in insomnia (55). Thus, insomnia may trigger a cascade of neuronal changes leading to central sensitization—which has long been considered a major contributor to chronic pain (56). The reverse influence of chronic pain on sleep is equally if not even more elusive (57) and might involve dysregulation of the hypothalamic-pituitary-adrenal axis (58). In addition, various behavioral and cognitive factors have been proposed to mediate the mutual influences of pain and poor sleep, including medication (59), catastrophizing (60), pre-sleep arousal (61), negative mood (62, 63) and attention (64). Clearly, more research is needed to better understand the interactions between insomnia and chronic pain, two highly prevalent conditions for which comorbidity is not unusual.

Some limitations of our study can be mentioned. First, we investigated the acute bidirectional effects of sleep quality and pain by means of subjective ratings. The real effects, as would be observed with repeated measurements of present pain and sleep quality, might differ from the perceived ones. On the other hand, subjective experience and conception about sleep and pain are not at all trivial as compared to objective indicators of their relationship. Even if the measured acute effects were purely subjective, the results reported here would still be of clinical relevance as they would in this case signify that dysfunctional beliefs about the consequences of poor sleep and pain were most exaggerated in people with severe symptomology and thus might represent an especially effective venue for interventions (46). Second, data were taken from a psychometric database which received input from volunteers. As a consequence, the sample might not precisely represent the general population. Third, participants could fill out different questionnaires at different times, possibly resulting in weaker observed associations than could be found with simultaneous completion. In light of these limitations and our novel results, future longitudinal studies with population-based samples are warranted so as to obtain more accurate symptom dynamics in the general population. Such insights may in turn facilitate future research on intervention strategies in line with the emerging “systems” approaches to psychopathology and psychotherapy.

## Author contributions

YW and TB collected and analyzed data. EV supervised the project. YW wrote the manuscript. All authors participated in the revision of the manuscript.

### Conflict of interest statement

The authors declare that the research was conducted in the absence of any commercial or financial relationships that could be construed as a potential conflict of interest.
